# Quality of Life in Breast Cancer Survivors: A Meta‐Analysis of Case‐Control Studies

**DOI:** 10.1002/pon.70398

**Published:** 2026-02-18

**Authors:** Yi‐Ran Huang, Hua‐Qing Xing, Yi‐Ran Yan, Yu‐Cheng Wang, Yuan Feng, Robert D. Smith, Zhaohui Su, Teris Cheung, Gabor S. Ungvari, Todd Jackson, Gang Wang, Qinge Zhang, Yu‐Tao Xiang

**Affiliations:** ^1^ Unit of Psychiatry Department of Public Health and Medicinal Administration Faculty of Health Sciences University of Macau Macao SAR China; ^2^ Centre for Cognitive and Brain Sciences University of Macau Macao SAR China; ^3^ Beijing Key Laboratory of Mental Disorders National Clinical Research Center for Mental Disorders & National Center for Mental Disorders Beijing Anding Hospital Capital Medical University Beijing China; ^4^ School of Public Health Southeast University Nanjing China; ^5^ School of Nursing Hong Kong Polytechnic University Hong Kong SAR China; ^6^ Section of Psychiatry University of Notre Dame Australia Fremantle Australia; ^7^ Division of Psychiatry School of Medicine University of Western Australia Perth Australia; ^8^ Department of Psychology University of Macau Macao SAR China

**Keywords:** breast cancer, cancer, case‐control studies, financial toxicity, insomnia, meta‐analysis, oncology, quality of life, survivorship, women's health

## Abstract

**Background:**

As the most prevalent malignancy among women, breast cancer has a potentially shattering impact on quality of life (QoL). However, studies comparing QoL between breast cancer survivors and cancer‐free peers have been inconsistent and little is known about possible moderators that contribute to inconsistent findings or specific QoL domains that affect breast cancer survivors most.

**Objectives:**

This meta‐analysis examined QoL differences between breast cancer survivors and controls without breast cancer (“controls” hereafter) across multiple QoL instruments and domains.

**Methods:**

We searched major international and Chinese databases and identified 36 eligible case‐control studies comprising 29,433 participants (12,261 survivors, 17,172 controls). Standardized mean differences were calculated using a random‐effects model; subgroup analyses and meta‐regression examined potential moderators.

**Results:**

QoL impairments varied by assessment instrument. Medical Outcomes Study Short Form surveys showed lower physical component scores in breast cancer survivors (moderate effect size, SMD = −0.52) and small deficits in physical function, emotional role limitations, mental components, and general health domains. The European Organization for Research and Treatment of Cancer questionnaire revealed lower scores in insomnia (SMD = 0.80) and financial difficulties (SMD = 0.77). Regarding the Functional Assessment of Cancer Therapy‐General, breast cancer survivors displayed comparatively large impairments across emotional, functional, and physical well‐being domains. Short‐term survivors (≤ 5 years post‐diagnosis) experienced significantly greater deficits than long‐term survivors did in physical role limitations and mental health domains.

**Conclusions:**

Breast cancer survivors experience lower QoL than controls do, particularly in physical and emotional domains. This meta‐analysis highlights the importance of developing effective interventions targeting specific QoL domains at different survivorship stages.

## 1 Introduction

Breast cancer is the most common malignancy among women globally [1], and accounts for an estimated 2.3 million incident cases annually [2]. In recent years, advances in early detection and therapeutic interventions have markedly enhanced prognoses for breast cancer, with 5‐year survival rates around 90% in high‐income countries [3, 4, 5]. Because these achievements have increased the population of long‐term survivors, the focus of research has shifted to exploring the impact of cancer and its therapies on quality of life (QoL) [6]. Despite improved survival, breast cancer survivors frequently experience significant psychological morbidities, including anxiety, depression, and post‐traumatic stress disorder (PTSD), which could lower QoL [7].

QoL is defined as an individual's subjective evaluation of their well‐being within cultural and value‐based contexts [8], which traditionally include physical, psychological, social and functional domains [9]. Unlike disease‐specific outcomes, QoL assessments offer a comprehensive view of patients' overall functioning and are indispensable for comprehensive mental health evaluations and care planning. Among breast cancer survivors, QoL is determined by diverse factors, including treatment sequelae, fear of recurrence, body image disturbances, occupational and social role disruptions and financial difficulties [10, 11, 12, 13, 14].

Despite growing recognition of QoL's importance, previous research comparing QoL between breast cancer survivors and breast cancer‐free peers has reported conflicting findings. One challenge for understanding QoL differences between breast cancer survivors versus peers without breast cancer concerns the lack of standardization in methodologies used to estimate QoL. Methodological inconsistencies include variability in QoL assessment tools, post‐diagnostic intervals, and sociocultural influences on well‐being perceptions. Widely employed instruments such as the World Health Organization Quality of Life Assessment (WHOQOL) [8], Medical Outcomes Study Short Form Health Survey (SF‐36/30/12; “SF” hereafter) [15], and EuroQol 5‐Dimension Questionnaire (EQ‐5D) [16] have demonstrated distinct psychometric properties and QoL domain content, as have cancer‐specific QoL measures such as the Functional Assessment of Cancer Therapy‐General (FACT‐G) [17] and European Organization for Research and Treatment of Cancer Quality of Life Questionnaire‐Core 30 (EORTC QLQ‐C30) [18]. This instrument diversity complicates cross‐study comparisons and possibly obscures domain‐specific QoL deficits.

Apart from instrument variability, additional confounding factors include clinical and sociodemographic variables. Treatment modalities (i.e., mastectomy vs. breast‐conserving surgery, chemotherapy regimens) [19] and clinical factors such as post‐diagnosis duration [20] can significantly influence QoL trajectories. In addition, age has emerged as a potentially important moderator, with younger survivors often exhibiting higher psychosocial vulnerability compared to older survivors, due to disruptions in fertility, career trajectories, and caregiving dynamics [21]. Furthermore, marital status and educational attainment may modulate adaptation processes [22], reflecting complex interactions between biological, psychological, and social influences.

Previous meta‐analyses have typically focused on QoL changes following specific interventions or restricted their focus to limited QoL domains with no direct comparisons between breast cancer survivors and breast cancer‐free controls [23]. A small meta‐analysis of 6 studies did not find significant QoL differences between survivors versus controls with no history of breast cancer [24]. However, the reliance on composite scores from heterogeneous instruments may have masked domain‐specific disparities. Furthermore, the exclusion of non‐English language databases in earlier reviews [24, 25, 26] may have neglected culturally mediated QoL variations. Given several recently published relevant studies, an updated meta‐analysis incorporating both English and Chinese databases is warranted to elucidate a comprehensive summary of QoL experiences for breast cancer survivors versus cancer‐free controls.

To address these research gaps, this meta‐analysis compared QoL between breast cancer survivors and healthy controls across domains and instruments using standardized effect size metrics, We also explored potential clinical and sociodemographic moderators (i.e., continents of study sites, study publication year, post‐diagnostic duration, and survivor mean age, education experiences, and marital status) of QoL group differences.

## 2 Methods

### 2.1 Search Strategy

A systematic search and screen of published evidence was conducted independently by three researchers (YRH, HQX and YRY) according to Preferred Reporting Items for Systematic Reviews and Meta‐Analyses (PRISMA) guidelines [27]. The protocol was pre‐registered with the International Prospective Register of Systematic Reviews (PROSPERO; Registration Number: CRD420251004739). No protocol amendments were made after registration. Four international databases (PubMed, Web of Science (WoS), EMBASE, and PsycINFO) and two Chinese databases (China National Knowledge Infrastructure (CNKI) and Wanfang) were searched from their dates of inception to December 12, 2024. The search terms were as follows: TS = [(“Breast Neoplasms” OR “Breast Carcinoma In Situ” OR “Carcinoma, Ductal, Breast” OR “Carcinoma, Lobular” OR “Hereditary Breast and Ovarian Cancer Syndrome” OR “Breast Tumor” OR “Tumor, Breast” OR “Breast Cancer” OR “Malignant Neoplasm of Breast” OR “Breast Malignant Neoplasm” OR “Mammary Cancer” OR “Human Mammary Neoplasm” OR “Human Mammary Carcinoma” OR “Mammary Carcinoma, Human” OR “Carcinoma, Human Mammary”)] AND [TS = (“Quality of Life” OR “QoL” OR “Life Quality”)] AND [TS = (“case control study”) OR “case‐control study” OR (case* AND control*)]. Reference lists from previous review articles were also checked for additional studies.

### 2.2 Inclusion and Exclusion Criteria

The selection criteria were determined based on the PICOS acronym: (a) Participants: Study‐defined survivors with breast cancer; (b) Intervention: Not applicable (NA); (c) Comparison: controls who did not have a history of breast cancer from comparable community or population‐based samples (“controls” hereafter); (d) Outcomes: Quality of Life (QoL) as measured by validated standard instruments such as the WHOQOL‐BREF, SF, EQ‐5D, EORTC QLQ‐C30 and FACT‐G; (e) Study design with accessible data: Cross sectional case‐control studies that compared QoL between breast cancer survivors versus controls. Those studies with special populations, such as institutionalized survivors or veterans, were excluded.

### 2.3 Data Extraction

Titles and abstracts of studies were independently checked by the three researchers, followed by full‐text screening for eligibility. Any disagreements between the three researchers that could not be addressed through discussion were resolved in consultation with a senior researcher (YTX). Relevant data were extracted independently by the same researchers using a standardized data extraction form. The following information was extracted: (a) study characteristics: first author, publication year, country/region, sampling method, and study design; (b) participant characteristics: total sample size, sample sizes for breast cancer survivors and controls, mean age, gender distribution, education level, marital status, and time since diagnosis. For time since diagnosis, we extracted the mean or range of post‐diagnostic years whenever these were reported. Furthermore, we classified studies into short‐term (≤ 5 years since diagnosis) and long‐term (> 5 years) survivorship according to the average or minimum time since diagnosis reported in included studies [28, 29]. We also extracted data on clinical characteristics of breast cancer survivors where available, including disease stage, and receipt of specific treatments (e.g., surgery type, chemotherapy, radiotherapy, endocrine therapy, targeted therapy). (c) Outcome measures: specific QoL instruments used and relevant overall and domain‐specific QoL scores (means and standard deviations) were reported.

When multiple papers reported findings from the same dataset, only the study that reported the most comprehensive and complete data was included in the analysis. For studies that reported QoL data in other formats (i.e., median and interquartile range), data conversion was performed using appropriate mathematical formulas [30]. Alternatively, either the first or corresponding author was contacted to obtain additional required data not reported in the original article.

### 2.4 Assessment of Study Quality

The assessment of study quality was conducted by the same three researchers (YRH, HQX, and YRY) independently using the Newcastle‐Ottawa Scale (NOS) [31] that includes the following domains: selection, comparability, and outcome or exposure. The maximum total NOS score is 9, with higher scores indicating higher study quality. A total NOS score of 7 or above indicated high quality, a score from 4 to 6 indicated moderate quality, and a score below 4 indicated low quality [32, 33]. Any disagreements on quality scoring were resolved through discussion and consultation with the senior researcher (YTX) to achieve consensus.

### 2.5 Statistical Analysis

All statistical analysis were performed using the packages, “meta (version 7.0‐0)” [34] and “metafor (version 4.6‐0)” [35] in R (4.4.1) [36]. Studies using the same QoL instruments (i.e., SF, EORTC QLQ‐C30, WHOQOL‐BREF) were pooled, while analyses were conducted for each domain separately. Effect sizes were calculated using standardized mean differences (SMDs) with 95% confidence intervals (CIs) to compare differences in each QoL domain between breast cancer survivors and controls. Based on Cohen's guidelines, SMD values of 0.2, 0.5, and 0.8 represented small, moderate and large effect sizes, respectively [37].

Considering study differences in instrument tools and survey samples, a random‐effects model was applied to analyze the data [38]. Heterogeneity was assessed using *Q* statistics and I^2^ statistics; I^2^ ≥ 50% or *p* < 0.1 in *Q* statistics indicated significant heterogeneity [39]. To identify potential sources of heterogeneity, subgroup analyses were performed for categorical variables: (1) continent: North America versus South America versus Europe versus Asia; (2) median publication year: studies published before versus after 2015; (3) post‐diagnostic duration of 5 years or longer versus shorter than 5 years; and (4) QoL instrument: WHOQOL‐Bref versus.SF versus EORTCQLQ‐C30 on physical and social domains, SF versus EORTCQLQ‐C30 on psychological and general QoL domains. Meta‐regression analyses were conducted for continuous variables based on the study quality (NOS) score, mean age of survivors, sample size of patients, education level, and proportion of survivors who were married or had partners [40].

A sensitivity analysis was performed to explore the impact of studies individually on overall results. Potential publication bias was evaluated using Egger's test and funnel plots [41]. All outcomes analyzed were considered significant at *p* < 0.05 with two‐tailed tests.

## 3 Results

### 3.1 Literature Search and Study Selection

**FIGURE 1:**
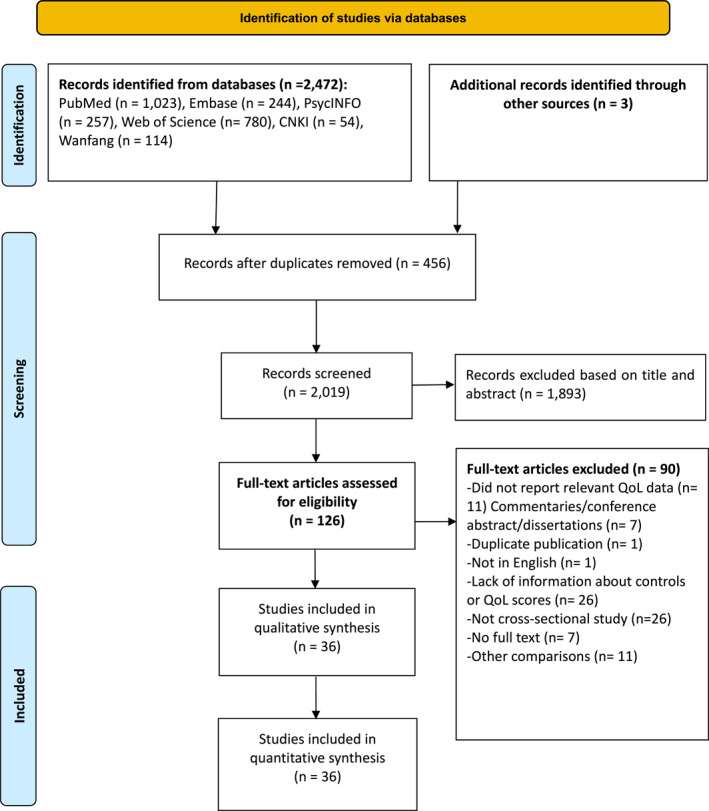
PRISMA flowchart.

Figure 1 illustrates the PRISMA flow diagram of the literature search and selection process. A total of 2472 records were retrieved. After removing duplicates and screening of titles and abstracts, 126 full text articles were assessed. Ultimately, 36 studies were included in the meta‐analysis.

Table 1 summarizes key characteristics of the included studies. In total, 29,433 participants were enrolled, including 12,261 breast cancer survivors and 17,172 controls. The mean participant age ranged from 28.3 to 65.0 years. Publication dates spanned from 2000 to 2024, with most studies conducted in North America, South America and Asia. NOS quality assessment scores ranged from 4 to 8, with 12 studies classified as high quality (33%) and 24 as moderate quality (67%). Detailed quality assessment data are available in Suporting Information S1: Table S1.

**TABLE 1:** Characteristics of studies included in this meta‐analysis.

No.	First author	Publication year	Study site (country)	Assessment of QOL	Breast cancer patient			Controls		NOS
					Age (mean ± SD)	*N*	Illness duration	Age (mean ± SD)	*N*	
1	Alaca, N., et al. [63]	2024	Turkey	EORTC QLQ‐C30	49.2 ± 10.7	140	NR	47.0 ± 7.5	72	7
2	Álvarez‐Salvago, F., et al. [64]	2024	Spain	EORTC QLQ‐C30	49.4 ± 8.1	80	≥ 5 years post diagnosis	49.4 ± 8.5	80	7
3	Amir, M., et al. [65]	2002	Israel	WHOQOL‐Bref	50.4 ± 6.2	39	≥ 5 years post diagnosis	NR	39	6
4	Awadalla, A. W., et al. [22]	2007	Sudan	WHOQOL‐Bref	43.0 ± 10.0	117	≥ 1 year post diagnosis	44.6 ± 11.7	177	6
5	Benton, M. J., et al. [66]	2019	U.S.	VPS	57.0 ± 2.3	23	6.4 ± 1.4 post diagnosis	49.0 ± 1.1	23	4
6	Boehmer, U., et al. [67]	2015	U.S.	SF‐12	51.6 ± 8.8	85	1–10 years post diagnosis	50.9 ± 9.1	85	6
7	Bøhn, S. K. H., et al. [68]	2024	Norway	EORTC QLQ‐C30	58.2 ± 11.2	4117	≥ 21 days post diagnosis	59.1 ± 11.2	2911	7
8	Broeckel, J. A., et al. [69]	2000	U.S.	SF‐36	51.6 ± 11.1	61	NR	51.5 ± 11.3	59	7
9	Champion, V. L., et al. [21]	2014	U.S.	SF‐36	NR	1012	3–8 years post diagnosis	NR	348	6
10	Chetrit, A., et al. [70]	2021	Israel	SF‐36	65.0 ± 10.3	250	8–12 years post diagnosis	65.3 ± 10.5	250	8
11	Claus, E. B., et al. [71]	2006	U.S.	SF‐36	55.8 ± 10.5	795	> 5 years post diagnosis	55 ± 11.7	702	8
12	Cordova, M. J., et al. [72]	2001	U.S.	SF‐20	54.7 ± 12.1	70	≤ 5 years post diagnosis	54.7 ± 13.2	70	6
13	de Larrea‐Baz, N. F., et al. [73]	2020	Spain	SF‐36	50.3 ± 9.5	896	NR	49.8 ± 9.4	890	7
14	Emre, N., et al. [56]	2024	Turkey	SF‐36	50.4 ± 7.1	125	NR	48.1 ± 10.1	125	6
15	Fenlon, D., et al. [74]	2013	UK	SF‐36	58.7 ± 10.9	247	NR	56.6 ± 8.8	274	4
16	Gudbergsson, S. B., et al. [75]	2007	Norway	SF‐12	52.8 ± 6.6	208	≤ 5 years post diagnosis	51.3 ± 5.9	208	7
17	Helgeson, V. S., et al. [76]	2005	U.S.	SF‐36	54.0	304	> 5 years post diagnosis	53.2	187	7
18	Hermelink, K., et al. [77]	2015	Germany	EORTC‐QLQ‐CF	50.4 ± 9.1	166	Newly diagnosis	52.6 ± 7.8	60	7
19	Hodgson, J. H., et al. [78]	2003	U.S.	SF‐36	58.4 ± 7.5	20	NR	61.7 ± 8.3	20	5
20	Kang, K. D., et al. [79]	2017	Korea	WHOQOL‐Bref	48.1 ± 7.0	76	NR	45.5 ± 8.0	44	6
21	Klein, D., et al. [80]	2011	France	EORTC QLQ‐C30, SF‐36	64.1	652	> 5 years post diagnosis	63.3	1188	8
22	Langer, D., et al. [81]	2023	Israel	FACT‐G	47.9 ± 8.3	140	1–10 years post diagnosis	49.0 ± 7.5	61	4
23	Liu, S. X., et al. [82]	2021	China	EORTC QLQ‐C30	NR	75	NR	NR	75	4
24	O'Sullivan, M. B., et al. [43]	2001	Ireland	SF‐36	NR	120	> 5 years post diagnosis	NR	284	5
25	Palomo‐López, P., et al. [83]	2017	Spain	FHSQ	53.6 ± 9.2	100	NR	51.0 ± 8.8	100	5
26	Ribeiro, I. L., et al. [42]	2019	Chile	SF‐36	50.2 ± 9.8	21	NR	50.7 ± 10.1	21	6
27	Schleife, H., et al. [84]	2014	Germany	EORTC QLQ‐C30	56.4 ± 10.5	107	NR	56.1 ± 13.9	880	6
28	Surbhi, H., et al. [85]	2022	India	WHOQOL‐Bref	NS	50	NR	NR	50	4
29	Tchen, N., et al. [86]	2003	Canada	FACT‐G	48.0 ± 8.3	100	NR	47.0 ± 9.0	100	6
30	Tolentino, G. P., et al. [87]	2010	Brazil	SF‐36	48.8 ± 8.9	22	≤ 2 years post diagnosis	45.5 ± 7.4	22	6
31	Tran, T. X. M., et al. [20]	2023	Korea	EQ‐5D	57.3 ± 8.5	273	9–16 years post diagnosis	57.2 ± 8.6	819	7
32	Von Ah, D. M., et al. [88]	2012	U.S.	SF‐36	57.3 ± 8.4	62	2–10 years post diagnosis	52.2 ± 15.4	78	6
33	Yabroff, K. R., et al. [89]	2007	U.S.	HALex	NR	920	NR	NR	4000	6
34	Yu, J., et al. [90]	2018	Korea	EQ‐5D	50.6 ± 8.2	686	NR	NR	2744	5
35	Zhang, J. H., et al. [91]	2011	China	EORTC QLQ‐C30	28.3 ± 7.7	90	NR	30.4 ± 6.8	70	6
36	Zhang, F. Y., et al. [92]	2008	China	GQ	34.9 ± 6.7	67	NR	35.3 ± 6.4	56	6

*Note:* All controls were recruited from community or population‐based samples. In the majority of studies, controls were matched to breast cancer survivors; in a small number of studies (study No.5, 23, 24, 28), matching procedures were not clearly described, although controls were still drawn from similar underlying populations.

Abbreviations: NOS, newcastle‐ottawa scale; SD, standard deviation.

### 3.2 QOL Measurement Scales and Comparison

Meta‐analyses were conducted for 32 out of the 36 included studies based on five QoL instruments (i.e., SF, EORTC QLQ‐C30, WHOQOL‐BREF, FACT‐G, and EQ‐5D). Four other studies were not included in the meta‐analysis due to the use of different QoL instruments. In general, there were significant differences between breast cancer survivors and controls regarding specific QoL domains of the SF, EORTC QLQ‐C30 and FACT‐G as well as the global EQ‐5D assessment.

**FIGURE 2:**
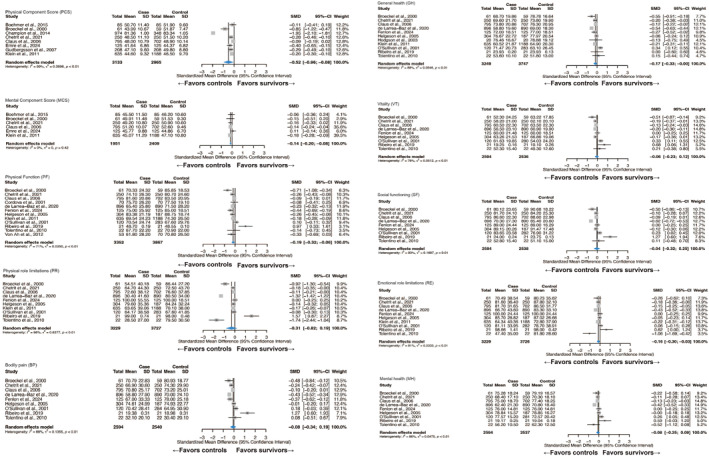
QOL comparison between breast cancer survivors and control groups using SF measures. CI, confidence interval; QOL, quality of life; SF, 36/20/12‐item Short Form Health Survey; SMD, standard mean difference. Negative SMD values indicate lower QoL in survivors compared to controls.

Analyses of 17 studies based on SF assessments (Figure 2) showed that compared with controls, breast cancer survivors had significantly poorer QoL in the Physical Component domain (SMD = −0.52, 95% CI: −0.96, −0.08; I^2^ = 98.6%) based on a moderate effect size difference. Survivors also scored lower on the Physical Function (SMD = −0.19, 95% CI: −0.32, −0.06; I^2^ = 70.8%), Emotional Role Limitations (SMD = −0.16, 95% CI: −0.30, −0.03; I^2^ = 81.3%), Mental Component (SMD = −0.14, 95% CI: −0.20, −0.08; I^2^ = 0.0%) and General Health domains (SMD = −0.17, 95% CI: −0.33, −0.00; I^2^ = 87.6%), each of which was characterized by a small effect size difference.

**FIGURE 3:**
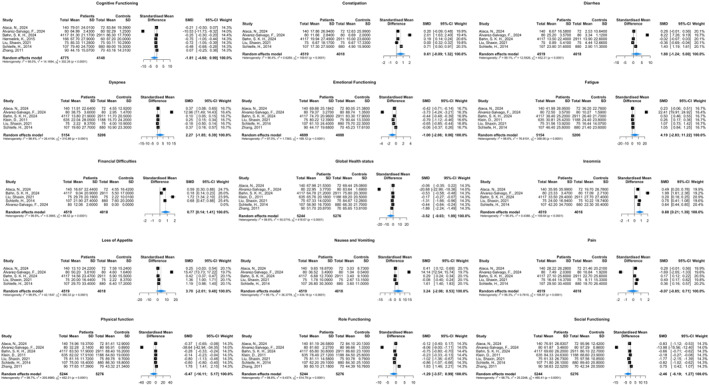
QOL comparison between breast cancer survivors and control groups using EORTC QLQ‐C30. CI, confidence interval; EORTC QLQ‐C30, The European Organization for Research and Treatment of Cancer Quality of Life Questionnaire; QOL, quality of life; SMD, Standard Mean difference. Higher scores on the functional scales indicate better health, while higher scores on the symptom scales indicate more symptom burden.

Analyses of eight studies based on EORTC QLQ‐C30 assessments (Figure 3) showed only two significant group differences on QoL dimensions. Breast cancer survivors reported poorer QoL on the Financial Difficulties domain (SMD = 0.77, 95% CI: 0.14, 1.41; I^2^ = 96.6%) and Insomnia domain (SMD = 0.80, 95% CI: 0.21, 1.39; I^2^ = 96.4%) compared to controls, with moderate and large effect size differences, respectively.

Based on four studies using the WHOQOL‐BREF scale, there were no significant differences between breast cancer survivors and controls across Physical, Psychological, Social, and Environmental QoL domains (Supporting Information S1: Figure S1). Two studies using the FACT‐G were included in the meta‐2analysis; results showed significant group differences in Emotional Well‐Being (SMD = −0.84, 95% CI: −1.06, −0.63; I^2^ = 0.0%), Functional Well‐Being (SMD = −1.08, 95% CI: −1.42, −0.74; I^2^ = 59.2%) and Physical Well‐Being domains (SMD = −1.29, 95% CI: −1.52, −1.07; I^2^ = 0.3%), as well as Total QoL Score (SMD = −1.10, 95% CI: −1.31, −0.88; I^2^ = 0.0%); effect size differences were large and reflected comparatively poorer functioning for breast cancer survivors (Supporting Information S1: Figure S2). Two studies using the EQ‐5D also indicated lower scores in breast cancer survivors (vs. controls) (SMD = −0.35, 95% CI: −0.53, −0.18; I^2^ = 77.9%) (Supporting Information S1: Figure S3).

### 3.3 Subgroup and Meta‐Regression Analyses

Results of subgroup analyses are presented in Table 2. In the SF assessments, survivors with long‐term survival (> 5 years) and short‐term survival (≤ 5 years) time exhibited significant differences in Physical Role Limitations and Mental Health. Specifically, compared to long‐term survivors, short‐term survivors exhibited significantly poorer QoL, with marked deficits in Physical Role Limitations (SMD = −1.38 vs. −0.14) and Mental Health domain (SMD = −0.42 vs. −0.01). Notably, however, a substantial proportion of studies did not report time since diagnosis. Therefore, while our findings showed more pronounced impairments in physical role limitations and mental health among short‐term survivors, the influence of post‐diagnosis duration differences on various QoL differences may have been underestimated. In subgroup analyses for the EORTC QLQ‐C30 assessment, continent and publication year were not significant moderators of group differences (Suporting Information S1: Table 2). Results of meta‐regression analyses for SF indexes are presented in Table 3. Higher study quality was significantly associated with lower Vitality QoL for breast cancer survivors (*β* = −0.11, *z* = −2.05, *p* = 0.04) but there were no other significant moderators.

**TABLE 2:** Subgroup analyses of QOL between breast cancer survivors and controls.

Subgroup	Domain	Category	No. of studies	SMD (95% CI)	I^2^	*p* value within subgroup	*p* value across subgroups
Continent	Physical component score	North America	4	−0.7521 (−1.6203, 0.1162)	99.30%	< 0.01	0.2639
		Europe	3	−0.2533 (−0.3611, −0.1455)	20.50%	0.28	
	Mental component score	North America	3	−0.1360 (−0.2290, −0.0430)	0.00%	0.88	0.6259
		Europe	2	−0.0620 (−0.3449, 0.2209)	78.40%	0.03	
	Physical functioning	North America	5	−0.2597 (−0.4561, −0.0633)	68.40%	0.01	0.4616
		Europe	4	−0.1843 (−0.3736, 0.0051)	74.40%	< 0.01	
		South America	2	0.4062 (−0.6830, 1.4954)	83.90%	0.01	
	Physical role limitations	North America	3	−0.3598 (−0.8550, 0.1354)	88.10%	< 0.01	0.9805
		Europe	4	−0.3974 (−1.0104, 0.2155)	99.00%	< 0.01	
		South America	2	−0.0827 (−3.3283, 3.1628)	97.70%	< 0.01	
	Bodily pain	North America	3	−0.1408 (−0.3448, 0.0632)	60.90%	0.08	0.3713
		Europe	3	−0.2112 (−0.5918, 0.1693)	92.40%	< 0.01	
		South America	2	0.6639 (−0.4952, 1.8229)	85.20%	< 0.01	
	General health	North America	4	−0.1700 (−0.3491, 0.0091)	46.60%	0.13	0.5584
		Europe	4	−0.1771 (−0.5342, 0.1800)	95.10%	< 0.01	
		South America	2	0.0776 (−0.3455, 0.5006)	0.00%	0.73	
	Vitality	North America	3	−0.1675 (−0.2592, −0.0758)	47.10%	0.15	0.0247
		Europe	3	0.0314 (−0.2816, 0.3444)	90.40%	< 0.01	
		South America	2	0.4351 (−0.0273, 0.8976)	13.60%	0.28	
	Social functioning	North America	3	−0.1579 (−0.3137, −0.0020)	55.40%	0.11	0.3601
		Europe	3	−0.1397 (−0.6498, 0.3703)	96.90%	< 0.01	
		South America	2	0.6794 (−0.4581, 1.8170)	84.60%	0.01	
	Emotional role limitations	North America	3	−0.1343 (−0.2204, −0.0481)	0.00%	0.48	0.9649
		Europe	4	−0.1648 (−0.3873, 0.0577)	88.50%	< 0.01	
		South America	2	−0.2159 (−1.8617, 1.4299)	92.70%	< 0.01	
	Mental health	North America	3	−0.1048 (−0.1959, −0.0137)	0.00%	0.39	0.9528
		Europe	3	−0.0635 (−0.4651, 0.3380)	94.80%	< 0.01	
		South America	2	0.0308 (−1.0515, 1.1132)	84.10%	0.01	
Publication year	Physical component score	≥ 2015	3	−0.2812 (−0.4112, −0.1513)	1.50%	0.36	0.2690
		< 2015	5	−0.6733 (−1.3563, 0.0097)	99.20%	< 0.01	
	Mental component score	≥ 2015	3	−0.0545 (−0.2235, 0.1144)	32.10%	0.23	0.2368
		< 2015	3	−0.1646 (−0.2333, −0.0959)	0.00%	0.84	
	Physical functioning	≥ 2015	8	−0.1848 (−0.3278, −0.0418)	62.60%	< 0.01	0.6464
		< 2015	4	−0.0541 (−0.5939, 0.4857)	81.50%	< 0.01	
	Physical role limitations	≥ 2015	6	−0.4597 (−0.9249, 0.0056)	86.40%	< 0.01	0.4775
		< 2015	4	−0.0149 (−1.1504, 1.1207)	98.70%	< 0.01	
	Bodily pain	≥ 2015	5	−0.0550 (−0.2458, 0.1358)	63.60%	0.03	0.8834
		< 2015	4	0.0008 (−0.7197, 0.7213)	88.70%	< 0.01	
	General health	≥ 2015	7	−0.0840 (−0.2883, 0.1202)	77.90%	< 0.01	0.1176
		< 2015	4	−0.3190 (−0.5309, −0.1071)	80.00%	< 0.01	
	Vitality	≥ 2015	5	−0.0661 (−0.3454, 0.2131)	82.00%	< 0.01	0.9947
		< 2015	4	−0.0673 (−0.2835, 0.1488)	68.50%	0.02	
	Social functioning	≥ 2015	5	−0.0709 (−0.2926, 0.1508)	70.60%	< 0.01	0.6896
		< 2015	4	0.0848 (−0.6464, 0.8160)	95.40%	< 0.01	
	Emotional role limitations	≥ 2015	6	−0.1523 (−0.2664, −0.0382)	66.90%	< 0.01	0.7050
		< 2015	4	−0.0804 (−0.4346, 0.2738)	86.90%	< 0.01	
	Mental health	≥ 2015	5	−0.0541 (−0.2535, 0.1453)	71.30%	< 0.01	0.9357
		< 2015	4	−0.0701 (−0.4040, 0.2638)	87.80%	< 0.01	
Post diagnostic duration (years)	Physical functioning	> 5 years	5	−0.1461 (−0.2530, −0.0392)	60.70%	0.04	0.3430
		≤ 5 years	3	−0.2132 (−0.3018, −0.1247)	0.00%	0.68	
	Physical role limitations	> 5 years	5	−0.1382 (−0.1970, −0.0793)	0.00%	0.88	**<** **0.01**
		≤ 5 years	2	−1.3790 (−1.6698, −1.0881)	26.00%	0.25	
	Bodily pain	> 5 years	4	−0.0531 (−0.2124, 0.1062)	68.90%	0.02	0.4169
		≤ 5 years	2	−0.2597 (−0.7323, 0.2129)	64.50%	0.09	
	General health	> 5 years	5	−0.0631 (−0.2454, 0.1192)	81.70%	< 0.01	0.5802
		≤ 5 years	2	−0.2574 (−0.9211, 0.4064)	80.30%	0.02	
	Vitality	> 5 years	4	−0.0503 (−0.2814, 0.1809)	82.80%	< 0.01	0.7957
		≤ 5 years	2	−0.1052 (−0.4507, 0.2403)	45.20%	0.18	
	Social functioning	> 5 years	4	−0.0336 (−0.1759, 0.1088)	62.60%	0.05	0.4417
		≤ 5 years	2	−0.3175 (−1.0266, 0.3917)	82.60%	0.02	
	Emotional role limitations	> 5 years	5	−0.1356 (−0.2183, −0.0528)	43.70%	0.13	0.0863
		≤ 5 years	2	−0.6612 (−1.2560, −0.0664)	72.90%	0.05	
	Mental health	> 5 years	4	−0.0088 (−0.1744, 0.1567)	73.20%	0.01	**<** **0.0001**
		≤ 5 years	2	−0.4241 (−0.5167, −0.3314)	0.00%	0.75	
Instrument	Physical domain	SF	12	−0.1868 (−0.3171, −0.0566)	70.8%	< 0.01	**<** **0.0001**
		EORTC QLQ‐C30	7	−5.4714 (−16.1137, 5.1710)	98.7%	< 0.01	
		WHOQOL‐BREF	3	0.0331 (−1.0820, 1.1482)	97.2%	< 0.01	
		FACT‐G	2	−1.2931 (−1.5168, −1.0694)	0.3%	0.317	
	Psychological domain	SF	6	−0.1435 (−0.2042, −0.0828)	0.0%	0.42	0.3157
		WHOQOL‐BREF	3	0.2737 (−0.5389, 1.0864)	94.6%	< 0.01	
	Social domain	SF	9	−0.0367 (−0.3250, 0.2516)	93.3%	< 0.01	0.6476
		EORTC QLQ‐C30	7	−2.4582 (−6.1866, 1.2702)	98.7%	< 0.01	
		WHOQOL‐BREF	3	−0.1335 (−0.9220, 0.6551)	93.9%	< 0.01	
		FACT‐G	2	−0.0960 (−1.0966, 0.9046)	95.7%	< 0.01	
	General health	SF	11	−0.1663 (−0.3292, −0.0034)	87.6%	< 0.01	0.2330
		EORTC QLQ‐C30	7	−3.5191 (−9.0271, 1.9888)	98.6%	< 0.01	

*Note:* Bolded values represent *p* < 0.05. The continent, publication year and post‐diagnostic year subgroup analyses were performed using studies employing the SF scale only, whereas the Instrument subgroup analysis includes studies that used either the WHOQOL‐BREF, SF or the EORTC QLQ‐C30.

Abbreviations: CI, confidence interval; EORTC QLQ‐C30, The European Organization for Research and Treatment of Cancer Quality of Life Questionnaire; SF, Short‐Form Health Survey (SF)‐36/12/20 items; SMD, standardized mean difference; WHOQOL‐BREF, The short version of WHOQOL.

**TABLE 3:** Meta‐regression analyses of QOL outcome using SF measures.

Variable	Domain	Number of studies	Coefficient	Standard error	95% Lower	95% Upper	*z* value	*p* value
NOS score	Physical component score	8	0.3251	0.2433	−0.1519	0.8020	1.3359	0.1816
	Mental component score	6	−0.096	0.0504	−0.1947	0.0027	−1.906	0.0567
	Physical functioning	12	−0.016	0.0608	−0.1353	0.1032	−0.2633	0.7923
	Physical role limitations	10	−0.0794	0.2126	−0.4960	0.3373	−0.3734	0.7088
	Bodily pain	9	−0.0547	0.1142	−0.2785	0.1690	−0.4794	0.6317
	General health	11	−0.0572	0.0626	−0.1800	0.0656	−0.9129	0.3613
	Vitality	9	−0.1081	0.0527	−0.2113	−0.0049	−2.0536	**0.0400**
	Social functioning	10	−0.1203	0.1143	−0.3443	0.1037	−1.0525	0.2926
	Emotional role limitations	10	−0.0587	0.0511	−0.1589	0.0414	−1.1492	0.2505
	Mental health	9	−0.0773	0.0625	−0.1997	0.0451	−1.2377	0.2158
Mean age (years)	Physical component score	7	0.0105	0.0138	−0.0165	0.0374	0.761	0.4466
	Mental component score	6	−0.0094	0.0061	−0.0213	0.0025	−1.5542	0.1201
	Physical functioning	11	−0.0068	0.0135	−0.0332	0.0197	−0.5011	0.6163
	Physical role limitations	9	0.0373	0.0531	−0.0667	0.1413	0.7023	0.4825
	Bodily pain	8	−0.0253	0.0325	−0.0891	0.0385	−0.778	0.4366
	General health	10	0.0075	0.012	−0.0161	0.0310	0.6211	0.5345
	Vitality	8	0.0029	0.0064	−0.0098	0.0155	0.446	0.6556
	Social functioning	8	−0.0071	0.0363	−0.0783	0.0641	−0.1952	0.8453
	Emotional role limitations	9	0.0091	0.0138	−0.0181	0.0362	0.6542	0.5130
	Mental health	8	0.0126	0.015	−0.0167	0.0420	0.8434	0.3990
Sample size of survivors	Physical component score	8	−0.0008	0.0006	−0.0021	0.0005	−1.2486	0.2118
	Mental component score	6	−0.0001	0.0001	−0.0004	0.0001	−1.1220	0.2619
	Physical functioning	12	0.0000	0.0002	−0.0005	0.0005	−0.0604	0.9518
	Physical role limitations	10	−0.0005	0.0009	−0.0022	0.0011	−0.6384	0.5232
	Bodily pain	9	−0.0005	0.0004	−0.0013	0.0004	−1.0715	0.2839
	General health	11	−0.0003	0.0002	−0.0008	0.0002	−1.3119	0.1896
	Vitality	9	−0.0003	0.0003	−0.0009	0.0002	−1.1037	0.2697
	Social functioning	9	−0.0007	0.0004	−0.0015	0.0001	−1.7284	0.0839
	Emotional role limitations	10	−0.0003	0.0002	−0.0006	0.0000	−1.8788	0.0603
	Mental health	9	−0.0004	0.0002	−0.0008	0.0000	−2.0992	**0.0358**
Above high school degree (%)	Physical component score	7	0.0029	0.0021	−0.0013	0.0071	1.3695	0.1708
	Mental component score	6	−0.0001	0.0011	−0.0023	0.0022	−0.0576	0.9541
	Physical functioning	8	0.0014	0.0013	−0.0011	0.0039	1.0772	0.2814
	Physical role limitations	6	0.0089	0.0076	−0.0060	0.0237	1.1741	0.2404
	General health	6	0.0038	0.0027	−0.0015	0.0091	1.3950	0.1630
	Emotional role limitations	6	0.0025	0.0019	−0.0013	0.0062	1.2846	0.1989
Marital status (%)	Physical component score	7	−0.0353	0.0285	−0.0911	0.0205	−1.2401	0.2149
	Physical functioning	9	−0.0016	0.0040	−0.0093	0.0061	−0.4049	0.6856
	Physical role limitations	7	0.0313	0.0309	−0.0293	0.0919	1.0129	0.3111
	Bodily pain	6	−0.0214	0.0077	−0.0365	−0.0062	−2.7615	**0.0058**
	General health	8	−0.0082	0.0078	−0.0235	0.0071	−1.0539	0.2919
	Vitality	6	−0.0022	0.0051	−0.0123	0.0078	−0.4350	0.6636
	Social functioning	6	−0.0114	0.0148	−0.0404	0.0177	−0.7667	0.4433
	Emotional role limitations	7	0.0159	0.0129	−0.0094	0.0413	1.2314	0.2182
	Mental health	6	0.004	0.0121	−0.0197	0.0276	0.3270	0.7436

*Note:* Bolded values represent *p* < 0.05.

Abbreviations: NOS, newcastle‐ottawa Scale for cohort studies; SF, Short‐Form Health survey (SF)‐36/12/20 items.

### 3.4 Publication Bias and Sensitivity Analyses

Funnel plot and Egger's test analyses were used to assess publication bias (Supporting Information S1: Figure S4). Significant publication bias was detected in the Bodily Pain (*p* < 0.05) and Social Functioning (*p* < 0.05) domains of SF assessments. Sensitivity analyses with a “leave‐one‐out” method were performed in SF assessments (Supporting Information S1: Figure S5). The analysis identified two outlier studies: Ribeiro et al. [42] significantly influenced primary results for Bodily Pain and Physical Role Limitations while O'Sullivan [43] impacted outcomes for the Vitality and General Health domains.

## 4 Discussion

This is the largest meta‐analysis to date comparing QoL differences between breast cancer survivors and controls across various QoL instruments and domains. The results demonstrated different patterns of QoL impairment for breast cancer survivors versus controls, depending, in part, on QoL instrument used and particular QoL dimensions assessed. Specifically, survivors exhibited significant deficits on QoL domains of Physical Component, Physical Functioning, Emotional Role Limitations, Mental Component, and General Health based on SF assessments while FACT‐G assessments revealed substantial impairments for survivors on Emotional, Functional and Physical Well‐Being domains. In contrast, no significant group differences were observed on WHOQOL‐BREF QoL domains.

We observed significantly lower scores on Physical Functioning domains among breast cancer survivors compared to controls in line with a previous meta‐analysis of six studies [24]. However, Moshina et al.’s meta‐analysis aggregated total QoL scores across heterogeneous instruments, limiting insights into potential domain‐specific effect size disparities. Our meta‐analysis addressed this limitation through stratifying analyses by assessment tool and comparing domain‐level heterogeneity. Furthermore, the inclusion of both English and Chinese databases in this meta‐analysis enhanced cross‐cultural generalizability, toward addressing a critical gap in the existing literature.

Deficits in physical‐related QoL were observed in breast cancer survivors across studies using SF and FACT‐G measures but not studies that used the WHOQOL‐BREF. SF and FACT‐G findings corroborate previous research [44], that highlighted persistent physical limitations following breast cancer treatment. Possible mechanisms underlying these physical domain deficits are multifactorial. First, treatment sequelae, such as muscle stiffness, breast sensitivity and concentration difficulties, are associated with poorer physical function [44, 45]. Second, surgical and treatment interventions, including mastectomy, axillary lymph node dissection and adjuvant therapies, often cause long‐term physical impairments such as fatigue and lymphedema [26, 46, 47]. In tandem, these factors could lower physical‐related QoL.

Emotional well‐being impairments among survivors identified in studies that used SF and FACT‐G instruments, but not the WHOQOL‐BREF, may be due to intersecting psychosocial and biological pathways. Psychological distress, including fear of cancer recurrence and post‐treatment body image disturbances, persistently impacts self‐concept and emotional well‐being [11, 13]. Endocrine therapy‐induced menopausal symptoms and mood alterations, often mediated by estrogen deprivation, further attenuate emotional recovery [48]. Neuropsychological effects of chemotherapy, generally known as “chemo brain,” compound emotional distress by disrupting frontal lobe function and reducing brain volume [49, 50]. In addition, social determinants of QoL, including occupational limitations and relationship strain, amplify these challenges by diminishing self‐efficacy and increasing isolation [51].

Compared to previous findings [24], a notable discrepancy identified in this meta‐analysis was in relation to comparatively increased financial difficulties endorsed by breast cancer survivors based on the EORTC QLQ‐C30 assessments. While most traditional QoL instruments focus on physical, psychological, social and environmental impacts, the EORTC QLQ‐C30 emphasizes facets of cancer‐related life impact including financial implications. Our findings are consistent with those conceptualizing financial stress as a critical dimension of cancer survivorship [14]. For instance, one cohort study indicated over 20% of breast cancer survivors experienced financial hardship even after 5 years post‐diagnosis [52]. This finding holds particular relevance given rising cancer care costs, variable financial protections across healthcare systems in many countries, and implications breast cancer may have for continuing employment [53, 54].

The heavy burden of insomnia was also identified among breast cancer survivors based on EORTC QLQ‐C30 results of this meta‐analysis. Breast cancer survivors are more likely to experience sleep disturbances than the general population is, even after 5 years post‐treatment [55]. Similar findings have also been observed among short‐term survivors, wherein poorer sleep quality is correlated with lower QoL scores [56]. In particular, the large effect size difference observed in this meta‐analysis indicates sleep disruptions represent a persistent survivorship challenge for breast cancer survivors. Insomnia prevalence among menopausal women, a high‐risk group for breast cancer, may exacerbate this burden [57]. Furthermore, insomnia is strongly associated with depression and anxiety, which are common mental health challenges for cancer survivors, creating possible bidirectional relationships that amplify insomnia severity [58].

Inconsistent findings across QoL instruments in subgroup analyses merit particular consideration. While the SF, EORTC QLQ‐C30 and FACT‐G instruments identified relative impairments across multiple domains, the WHOQOL‐BREF did not find significant between group differences. This discrepancy likely reflects differences in instrument sensitivity, domain coverage and conceptual frameworks. Cancer‐specific tools, such as EORTC QLQ‐C30 and FACT‐G, may capture specific survivorship concerns more effectively than generic instruments such as the WHOQOL do [59]. This observation highlights the importance of instrument selection in survivorship research: cancer‐specific measures are more sensitive in identifying QoL issues salient for survivors and yield more clinically meaningful insights than generic QoL tools do [60].

Subgroup analyses found that short‐term survivors exhibited poorer performance in domains of physical role limitations and the mental health domain compared to long‐term survivors. This finding may reflect gradual adaptation to changes over time among survivors. Some theoretical models suggest that positive adaptation arises through self‐concept recalibration and coping strategies. For instance, familial support can foster resilience despite chronic illness [51, 61]. Early‐phase impairment in physical role limitations likely reflects the acute burden of treatment side effects, including fatigue, functional limitations and physical deconditioning; presumably early rehabilitation interventions could mitigate functional declines during this phase [62]. While time since diagnosis emerged as an important source of heterogeneity in this meta‐analysis, regrettably, many included studies failed to report post‐diagnostic durations or time ranges, precluding their inclusion in subgroup analyses. As a result, pooled estimates may have partially obscured differences between the acute treatment phase and longer‐term survivorship. Additionally, meta‐regression analyses found higher study quality based on NOS scores was correlated with lower Vitality in QoL among breast cancer survivors. It is not clear why a significant effect emerged for study quality and vitality but no other QoL domain. Given that this was the sole significant association observed out of the 10 uncorrected analyses run on study quality‐QoL relations, replications are needed to ensure this effect is meaningful and not a Type I error.

Sensitivity analyses identified two outlier studies that affected select findings. One study reported highly elevated pain and functional limitations for survivors [42] based on SF QoL dimensions of Bodily Pain and Physical Role Limitations. These findings may reflect the study's exclusive focus on breast cancer survivors who underwent breast surgery, axillary lymphadenectomy and sentinel lymph node biopsy. Primary results for SF QoL domains of Vitality and General Health were affected by another outlier study [43] which reported paradoxically higher QoL scores in the breast cancer group than the control group. Possibly this finding was due to scoring errors or reflected cultural and regional differences, as the sample was limited to Irish respondents [43].

## 5 Clinical Implications

Findings from this meta‐analysis have several important clinical implications. First, the identification of domain‐specific QoL impairments suggests the need for tailored interventions targeting physical functioning, emotional well‐being, financial concerns, and sleep quality. Second, financial difficulties experienced by breast cancer survivors highlight the necessity of integrating financial navigation support into standard care protocols, including insurance counseling and employment assistance. Third, given the substantial burden of insomnia identified in our analyses, systematic sleep assessments and evidence‐based sleep interventions should be incorporated into survivorship care plans. Fourth, differential QoL impacts observed between short‐term and long‐term survivors underscore the importance of dynamic survivorship care planning that evolves with changing patient needs. Finally, our findings regarding instrument sensitivity suggest that clinical assessments of breast cancer should preferentially employ cancer‐specific QoL measures to more accurately identify and monitor survivorship challenges.

## 6 Study Limitations

Strengths of this meta‐analysis included the addition of new case‐control studies involving a large number of breast cancer survivors, which increased the statistical power of analyses. Additionally, stratified analyses of QoL domains were conducted based on standardized instruments (i.e., SF, EORTC QLQ‐C30, FACT‐G), enabling novel insights into instrument‐ and domain‐specific impairment differences between survivors and controls. Moreover, by including studies from both English and Chinese databases, the study enhances cross‐cultural generalizability and strengthens applicability to diverse healthcare contexts.

Despite these strengths, several limitations should also be noted. First, apart from the SF and EORTC QLQ‐C30, subgroup analyses and meta‐regression could not be performed for studies using other QoL instruments (i.e., the WHOQOL‐BREF, FACT‐G, and EQ‐5D) due to limited study numbers. Second, subgroup and meta‐regression analyses on certain clinical factors associated with QoL, such as tumor stage, surgery type and treatments, could not be performed due to inconsistent reporting of these details between studies. Given their potential salience to QoL, future studies should fully report tumor data on stage, hormone receptor & HER2 status, and adjuvant chemotherapy/targeted therapies/endocrine therapies as a matter of course so that future reviews can elucidate their impact on survivor QoL in an empirically‐grounded way. Third, the self‐report nature of QoL instruments introduces potential reporting biases, including recall bias and response shift phenomena; incorporating significant other reports within future studies may help to address this concern. Finally, due to the cross‐sectional nature of included studies, causal inferences regarding relations between breast cancer survivorship and QoL trajectories could not be drawn.

## 7 Conclusion

In conclusion, this meta‐analysis revealed domain‐specific QoL impairments in breast cancer survivors that varied by assessment instrument, with notable deficits in physical functioning, emotional well‐being, financial stability and sleep quality among survivors compared to controls. The substantial impact of post‐diagnostic duration on select QoL domains highlights the dynamic nature of survivorship challenges, with greater impairments observed during early survivorship that were attenuated for long‐term survivors. Overall findings highlighted the need for full reporting of sample characteristics and intervention details in future studies as well as comprehensive rehabilitation services, financial navigation support, systematic sleep assessment, and dynamic survivorship care planning to improve QoL in breast cancer survivors.

## Author Contributions

Study design: Yuan Feng, Gang Wang, Qinge Zhang, Yu‐Tao Xiang. Data collection, analysis and interpretation: Yi‐Ran Huang, Hua‐Qing Xing, Yi‐Ran Yan, Yu‐Cheng Wang, Zhaohui Su, Teris Cheung, Gabor S. Ungvari. Drafting of the manuscript: Yi‐Ran Huang, Hua‐Qing Xing, Yu‐Tao Xiang. Critical revision of the manuscript: Robert D. Smith, Todd Jackson. Approval of the final version for publication: all co‐authors.

## Funding

The study was supported by Beijing High Level Public Health Technology Talent Construction Project (Discipline Backbone‐01‐028), the Beijing Municipal Science & Technology Commission (No. Z181100001518005), the Capital's Funds for Health Improvement and Research (CFH 2024‐2–1174) and the University of Macau (MYRG‐GRG2023‐00141‐FHS; CPG2025‐00021‐FHS).

## Ethics Statement

No ethical review was required. Data were used from previously published studies, where consent had already been obtained from the investigators.

## Conflicts of Interest

The authors declare no conflicts of interest.

## Supporting information


Supporting Information S1

## Data Availability

The data that support the findings of this study are available from the corresponding author upon reasonable request.
