# Supplementary material for: Quality of Life in Breast Cancer Survivors: A Meta‐Analysis of Case‐Control Studies
Source: Psychooncology. 2026 Feb 18;35(2):e70398. doi: 10.1002/pon.70398 (PMC12916082; doi:10.1002/pon.70398)
Supplement: Supplementary file 1 — Supporting Information S1 [file PON-35-e70398-s001.docx]

**Supplementary materials**

**Table S1.** Study quality using the Newcastle-Ottawa Scale

**Table S2.** Subgroup analyses of QOL between breast cancer survivors and controls using the EORTC QLQ-C30

**Figure S1.** QOL comparison between breast cancer survivors and control group using the WHOQOL-BREF

Note: QOL, Quality of life; WHOQOL-BREF, World Health Organization Quality of Life Brief Version; SMD, Standard Mean difference; CI, Confidence Interval

**Figure S2.** QOL comparison between breast cancer survivors and control group using the FACT-G

Note: QOL, Quality of life; FACT-G, Functional Assessment of Cancer Therapy-General; SMD, Standard Mean difference; CI, Confidence Interval

**Figure S3.** QOL comparison between breast cancer survivors and control group using EQ-5D

Note: QOL, Quality of life; SMD, Standard Mean difference; CI, Confidence Interval

**Figure S4.** Funnel plot of pooled SMD of QoL in breast cancer survivors using SF

Note: QOL, Quality of life; SMD, Standard Mean difference; SF, 36/20/12-Item Short Form Health Survey

**Figure S5.** Sensitivity analysis of pooled SMD of QoL in breast cancer

survivors using SF

Note: QOL, Quality of life; SMD, Standard Mean difference; SF, 36/20/12-Item Short Form Health Survey

**Supplementary Table 1.** Study quality using the Newcastle-Ottawa Scale

| No. | References | First author, Year | Total Score | Selection | | | | Comparability （Comparability of cases and controls on the basis of the design or analysis） | | Exposure | | |
| --- | --- | --- | --- | --- | --- | --- | --- | --- | --- | --- | --- | --- |
|  |  |  |  | 1) Is the patient case definition adequate (the inclusion criteria of PD group were clearly stated) | 2) Representativeness of the PD cases (consecutive or obviously representative *; potential bias or not stated) | 3) Selection of controls (community *; hospital controls; no description) | 4) Definition of controls (no history disease *; no description) | a) Study controls for age (Select the most important factor *.) | b) study controls for any additional factor *, i.e., educational level | 1) Ascertainment of exposure (secure record *; structured interview where blind to case/control status*; others) | 2) Same method of ascertainment for cases and controls (yes *; no) | 3) Non-Response rate (same rate for both groups *; no description) |
| 1 | (1) | Alaca, N., et al., 2024 | 7 | 1 | 1 | 1 | 1 | 1 | 0 | 1 | 1 | 0 |
| 2 | (2) | Álvarez-Salvago, F., et al., 2024 | 7 | 1 | 0 | 1 | 1 | 1 | 1 | 1 | 1 | 0 |
| 3 | (3) | Amir, M., et al., 2002 | 6 | 1 | 1 | 0 | 1 | 1 | 0 | 1 | 1 | 0 |
| 4 | (4) | Awadalla, A. W., et al., 2007 | 6 | 1 | 1 | 0 | 1 | 1 | 0 | 1 | 1 | 0 |
| 5 | (5) | Benton, M. J., et al., 2019 | 4 | 0 | 0 | 1 | 1 | 0 | 0 | 1 | 1 | 0 |
| 6 | (6) | Boehmer, U., et al., 2015 | 6 | 0 | 0 | 1 | 1 | 1 | 0 | 1 | 1 | 1 |
| 7 | (7) | Bøhn, S. K. H., et al., 2024 | 7 | 1 | 1 | 1 | 1 | 1 | 0 | 1 | 1 | 0 |
| 8 | (8) | Broeckel, J. A., et al., 2000 | 7 | 1 | 0 | 1 | 1 | 1 | 0 | 1 | 1 | 1 |
| 9 | (9) | Champion, V. L., et al., 2014 | 6 | 1 | 0 | 1 | 1 | 1 | 0 | 1 | 1 | 0 |
| 10 | (10) | Chetrit, A., et al., 2021 | 8 | 1 | 1 | 1 | 1 | 1 | 0 | 1 | 1 | 1 |
| 11 | (11) | Claus, E. B., et al., 2006 | 8 | 1 | 1 | 1 | 1 | 1 | 0 | 1 | 1 | 1 |
| 12 | (12) | Cordova, Matthew J., et al., 2001 | 6 | 1 | 0 | 0 | 1 | 1 | 0 | 1 | 1 | 1 |
| 13 | (13) | de Larrea-Baz, N. F., et al., 2020 | 7 | 1 | 1 | 1 | 1 | 1 | 0 | 1 | 1 | 0 |
| 14 | (14) | Emre, N., et al., 2024 | 6 | 1 | 0 | 1 | 1 | 1 | 0 | 1 | 1 | 0 |
| 15 | (15) | Fenlon, D., et al., 2013 | 4 | 1 | 0 | 0 | 1 | 0 | 0 | 1 | 1 | 0 |
| 16 | (16) | Gudbergsson, S. B., et al., 2007 | 7 | 1 | 1 | 1 | 1 | 1 | 0 | 1 | 1 | 0 |
| 17 | (17) | Helgeson, V. S., et al., 2005 | 7 | 1 | 1 | 1 | 1 | 1 | 0 | 1 | 1 | 0 |
| 18 | (18) | Hermelink, K., et al., 2015 | 7 | 1 | 1 | 1 | 1 | 1 | 0 | 1 | 1 | 0 |
| 19 | (19) | Hodgson, J. H., et al., 2003 | 5 | 1 | 0 | 0 | 1 | 0 | 0 | 1 | 1 | 1 |
| 20 | (20) | Kang, K. D., et al., 2017 | 6 | 1 | 0 | 0 | 1 | 1 | 0 | 1 | 1 | 1 |
| 21 | (21) | Klein, D., et al., 2011 | 8 | 1 | 1 | 1 | 1 | 1 | 0 | 1 | 1 | 1 |
| 22 | (22) | Langer, D., et al., 2023 | 4 | 0 | 0 | 0 | 1 | 1 | 0 | 1 | 1 | 0 |
| 23 | (23) | Liu, S. X., et al., 2021 | 4 | 1 | 0 | 0 | 1 | 0 | 0 | 1 | 1 | 0 |
| 24 | (24) | O'Sullivan, M. B., et al. 2001 | 5 | 1 | 1 | 0 | 1 | 0 | 0 | 1 | 1 | 0 |
| 25 | (25) | Palomo-López, P., et al., 2017 | 5 | 1 | 1 | 0 | 1 | 0 | 0 | 1 | 1 | 0 |
| 26 | (26) | Ribeiro, I. L., et al., 2019 | 6 | 1 | 1 | 0 | 1 | 1 | 0 | 1 | 1 | 0 |
| 27 | (27) | Schleife, H., et al., 2014 | 6 | 1 | 1 | 0 | 1 | 1 | 0 | 1 | 1 | 0 |
| 28 | (28) | Surbhi, H., et al., 2022 | 4 | 1 | 0 | 0 | 1 | 0 | 0 | 1 | 1 | 0 |
| 29 | (29) | Tchen, N., et al., 2003 | 6 | 1 | 0 | 1 | 1 | 1 | 0 | 1 | 1 | 0 |
| 30 | (30) | Tolentino, G. P., et al., 2010 | 6 | 1 | 0 | 1 | 1 | 1 | 0 | 1 | 1 | 0 |
| 31 | (31) | Tran, T. X. M., et al., 2023 | 7 | 1 | 1 | 1 | 1 | 1 | 0 | 1 | 1 | 0 |
| 32 | (32) | Von Ah, D. M., et al., 2012 | 6 | 1 | 0 | 1 | 1 | 1 | 0 | 1 | 1 | 0 |
| 33 | (33) | Yabroff, K. R., et al., 2007 | 6 | 1 | 0 | 1 | 1 | 1 | 0 | 1 | 1 | 0 |
| 34 | (34) | Yu, J., et al., 2018 | 5 | 1 | 0 | 0 | 1 | 1 | 0 | 1 | 1 | 0 |
| 35 | (35) | Zhang, J. H., et al., 2011 | 6 | 1 | 0 | 1 | 1 | 1 | 0 | 1 | 1 | 0 |
| 36 | (36) | Zhang, F. Y., et al., 2008 | 6 | 1 | 0 | 1 | 1 | 1 | 0 | 1 | 1 | 0 |

**Supplementary Table 2.** Subgroup analyses of QOL between breast cancer survivors and controls using the EORTC QLQ-C30

| Subgroup | Domain | Category | No. of studies | SMD (95% CI) | **I^2^** | P value within subgroup | P value across subgroups |
| --- | --- | --- | --- | --- | --- | --- | --- |
| Continent | Global health status | Europe | 5 | -4.3211 (-12.2285, 3.5864) | 98.80% | <0.01 |  |
|  |  | Asia | 2 | -1.5829 ( -2.1265, -1.0392) | 77.60% | 0.03 | 0.4983 |
|  | Physical function | Europe | 5 | -7.9058 (-22.7678, 6.9562) | 98.80% | <0.01 |  |
|  |  | Asia | 2 | 0.4891 (-2.0346, 3.0129) | 99.00% | <0.01 | 0.2751 |
|  | Role functioning | Europe | 5 | -1.9793 (-4.9240, 0.9655) | 98.80% | <0.01 |  |
|  |  | Asia | 2 | 0.4086 (-2.3839, 3.2011) | 99.20% | <0.01 | 0.2488 |
|  | Emotional functioning | Europe | 4 | -1.2947 (-2.8606, 0.2713) | 98.10% | <0.01 |  |
|  |  | Asia | 2 | -0.4208 (-1.1402, 0.2985) | 89.90% | <0.01 | 0.3203 |
|  | Cognitive functioning | Europe | 5 | -2.4143 (-6.3357, 1.5070) | 98.60% | <0.01 |  |
|  |  | Asia | 2 | -0.3231 (-1.0912, 0.4449) | 91.20% | <0.01 | 0.3050 |
|  | Social functioning | Europe | 5 | -3.2554 (-8.4409, 1.9301) | 98.90% | <0.01 |  |
|  |  | Asia | 2 | -0.5076 (-2.9721, 1.9568) | 99.00% | <0.01 | 0.3482 |
| Publication Year | Global health status | ≥2015 | 4 | -5.5927 (-15.3582, 4.1729) | 99.10% | <0.01 |  |
|  |  | <2015 | 3 | -0.8119 (-1.8304, 0.2065) | 97.40% | <0.01 | 0.3399 |
|  | Physical function | ≥2015 | 4 | -9.9282 (-28.5022, 8.6459) | 99.00% | <0.01 |  |
|  |  | <2015 | 3 | 0.3376 (-1.0813, 1.7564) | 98.40% | <0.01 | 0.2801 |
|  | Role functioning | ≥2015 | 4 | -2.4623 (-6.0935, 1.1689) | 98.80% | <0.01 |  |
|  |  | <2015 | 3 | 0.2394 (-1.3491, 1.8280) | 98.70% | <0.01 | 0.1815 |
|  | Emotional functioning | ≥2015 | 4 | -1.3313 (-2.8820, 0.2195) | 98.10% | <0.01 |  |
|  |  | <2015 | 2 | -0.3642 (-0.9431, 0.2147) | 89.70% | <0.01 | 0.2522 |
|  | Cognitive functioning | ≥2015 | 5 | -2.4618 (-6.3621, 1.4386) | 98.60% | <0.01 |  |
|  |  | <2015 | 2 | -0.2205 (-0.7565, 0.3155) | 88.00% | <0.01 | 0.2645 |
|  | Social functioning | ≥2015 | 4 | -4.2734 (-10.5629, 2.0162) | 99.00% | <0.01 |  |
|  |  | <2015 | 3 | -0.0909 (-0.9736, 0.7917) | 97.10% | <0.01 | 0.1968 |
|  | Fatigue | ≥2015 | 4 | 5.9981 (-4.6214, 16.6176) | 99.00% | <0.01 |  |
|  |  | <2015 | 2 | 0.6497 (-0.1192, 1.4187) | 97.80% | <0.01 | 0.3249 |
|  | Dyspnea | ≥2015 | 4 | 3.2787 (-2.9860, 9.5434) | 99.00% | <0.01 |  |
|  |  | <2015 | 2 | 0.2718 (0.1785, 0.3650) | 6.10% | 0.30 | 0.3469 |

Note: SMD = Standardized Mean Difference; CI = Confidence Interval; EORTC-QLQ-CF = The European Organisation for Research and Treatment of Cancer Quality of Life Questionnaire-Cognitive functioning.

**
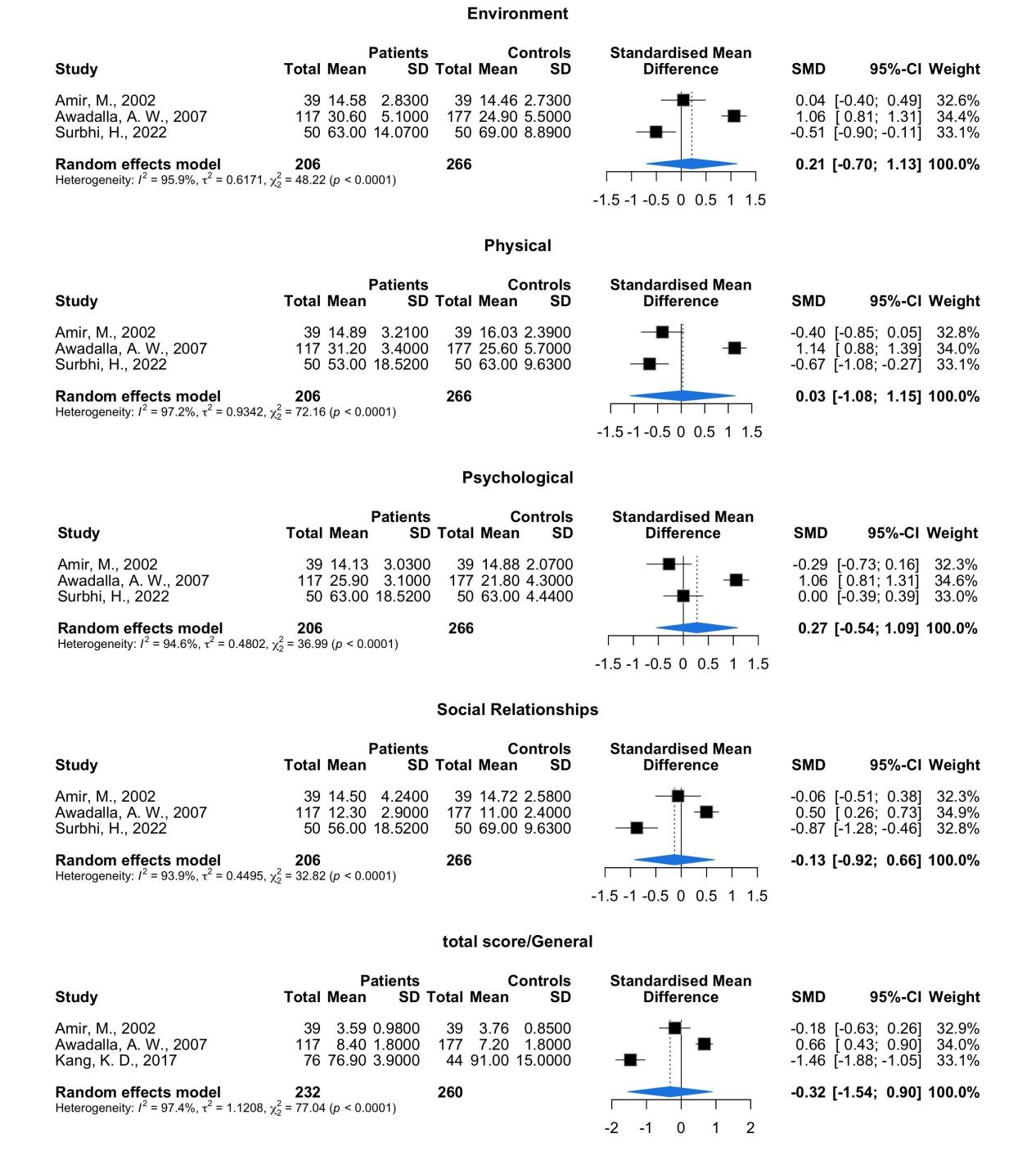
**

**Figure S1.** QOL comparison between breast cancer survivors and control group using the WHOQOL-BREF

Note: QOL, Quality of life; WHOQOL-BREF, World Health Organization Quality of Life Brief Version; SMD, Standard Mean difference; CI, Confidence Interval

**
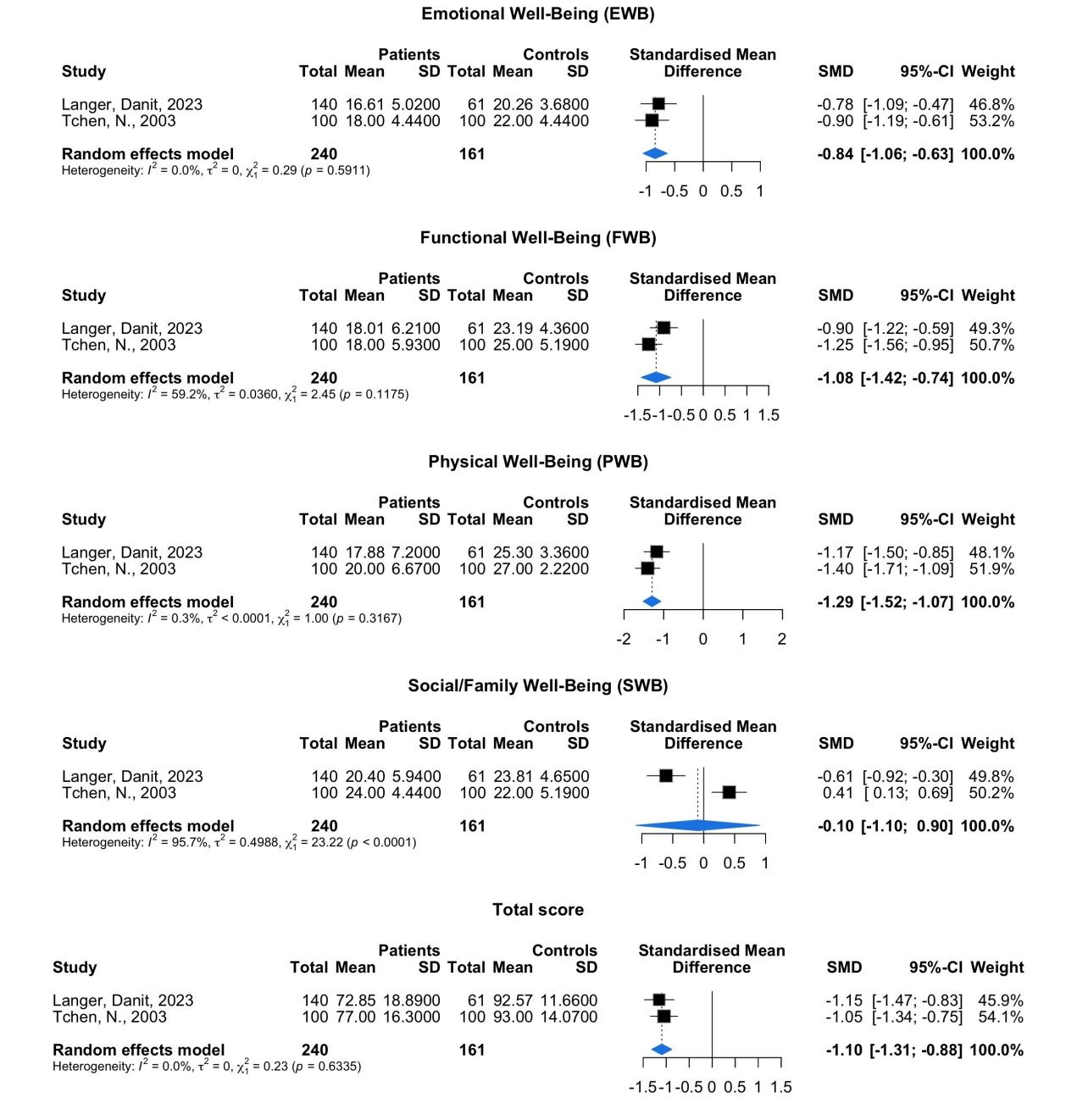
**

**Figure S2.** QOL comparison between breast cancer survivors and control group using the FACT-G

Note: QOL, Quality of life; FACT-G, Functional Assessment of Cancer Therapy-General; SMD, Standard Mean difference; CI, Confidence Interval

**
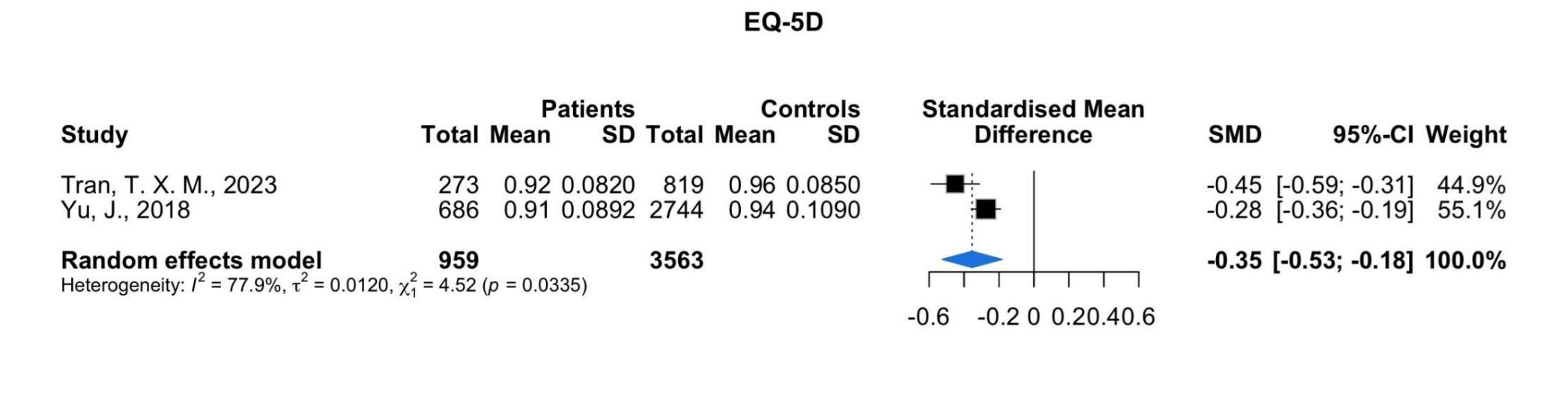
**

**Figure S3.** QOL comparison between breast cancer survivors and control group using EQ-5D

Note: QOL, Quality of life; SMD, Standard Mean difference; CI, Confidence Interval

**
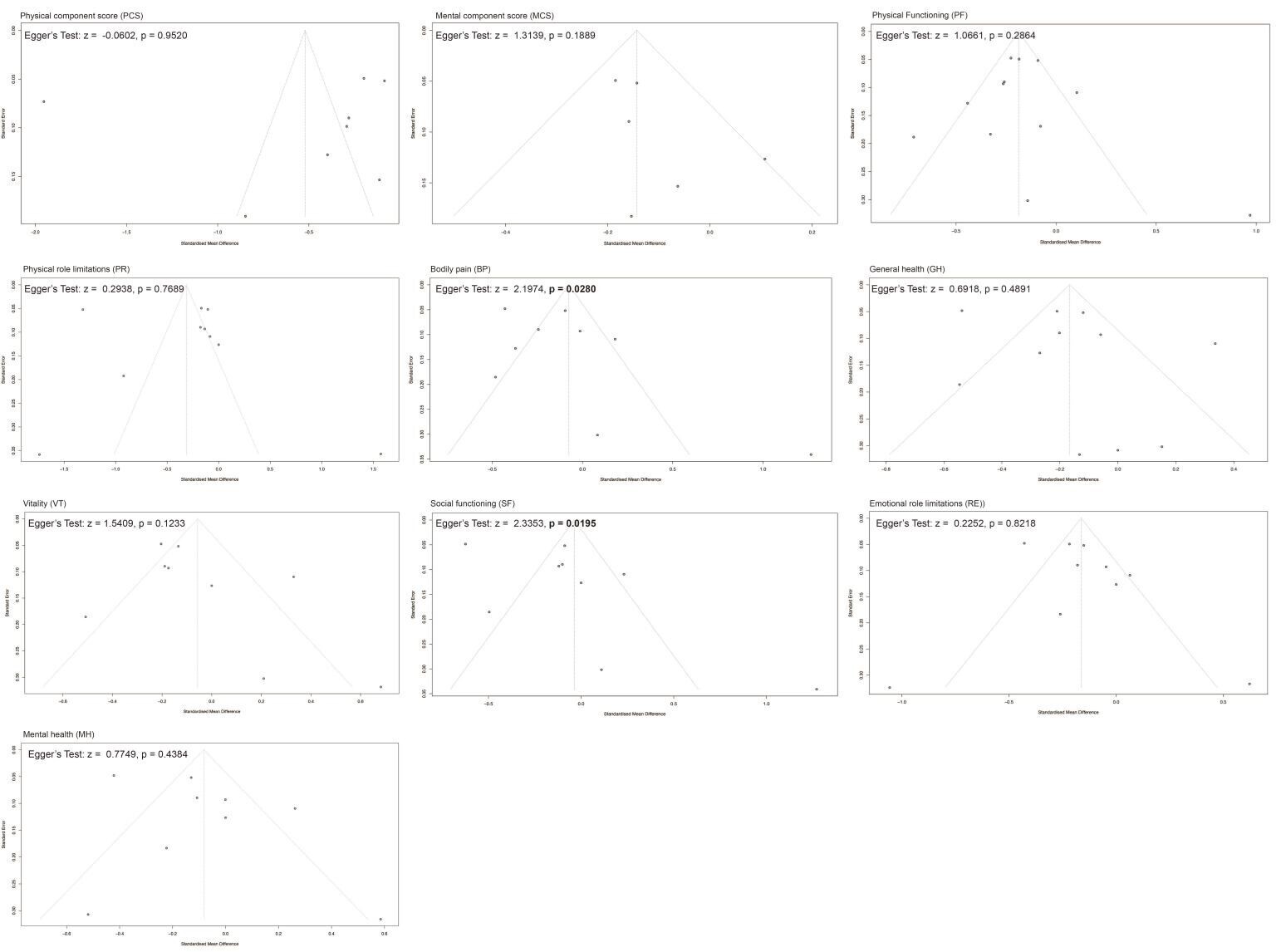
**

**Figure S4.** Funnel plot of pooled SMD of QoL in breast cancer survivors using SF

Note: QOL, Quality of life; SMD, Standard Mean difference; SF, 36/20/12-Item Short Form Health Survey

**
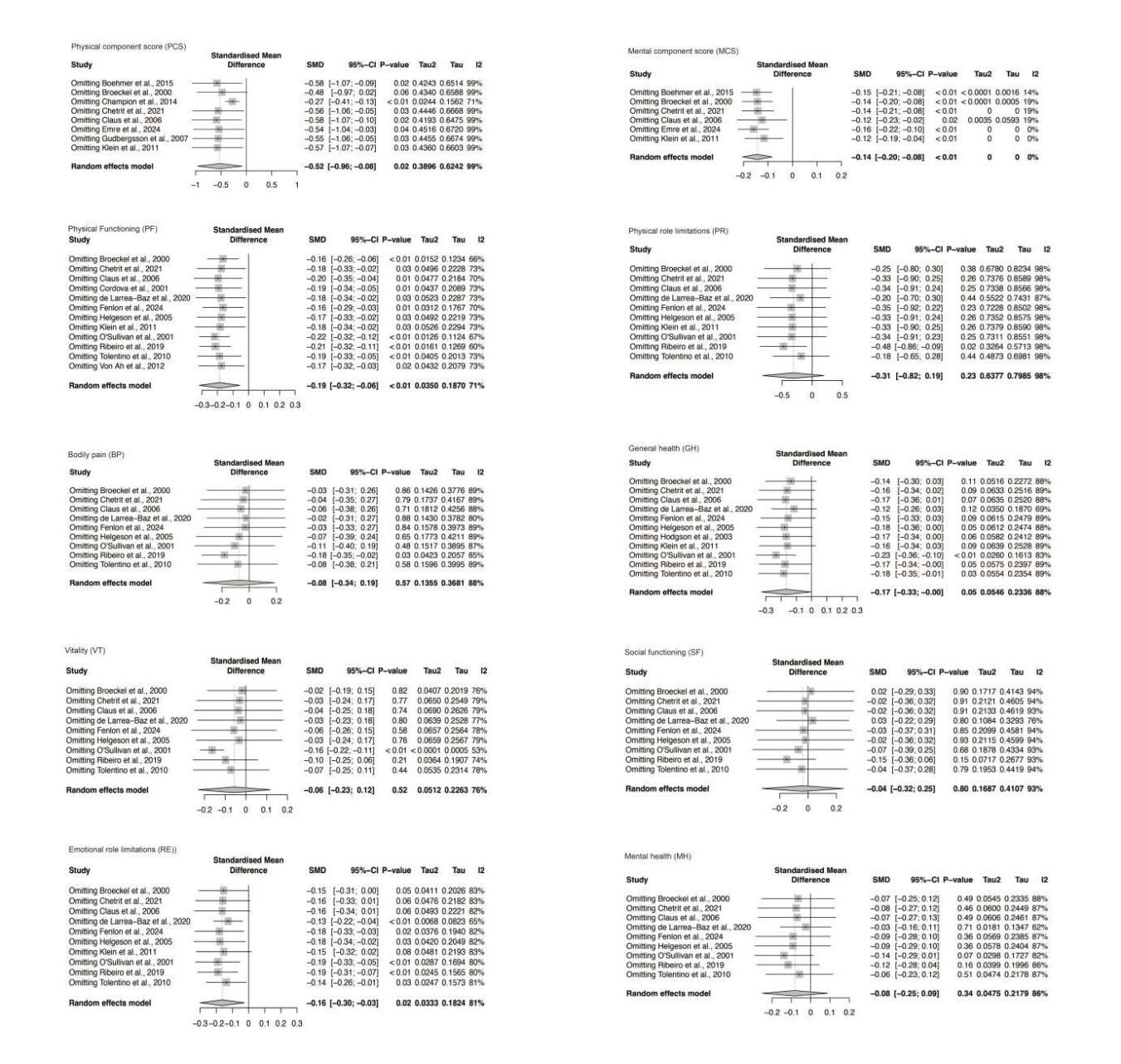
**

**Figure S5.** Sensitivity analysis of pooled SMD of QoL in breast cancer

survivors using SF measures

Note: QOL, Quality of life; SMD, Standard Mean difference; SF, 36/20/12-Item Short Form Health Survey

**References**

1. Alaca N, Karayazi KT, Arslan DC, Karakus MB, Uras C. Kinesiophobia, physical activity levels and barriers in breast cancer patients, survivors, and healthy controls: A case-control analysis. Journal of the Pakistan Medical Association. 2024;74(8):1428-36.

2. Álvarez-Salvago F, Gutiérrez-García P, Molina-García C, Atienzar-Aroca S, Jiménez-García JD, Aibar-Almazán A, et al. Is it really over when it is over? physical, mental and emotional health status of long-term breast cancer survivors compared to healthy matched controls. Support Care Cancer. 2024;32(10):641.

3. Amir M, Ramati A. Post-traumatic symptoms, emotional distress and quality of life in long-term survivors of breast cancer: a preliminary research. J Anxiety Disord. 2002;16(2):195-206.

4. Awadalla AW, Ohaeri JU, Gholoum A, Khalid AO, Hamad HM, Jacob A. Factors associated with quality of life of outpatients with breast cancer and gynecologic cancers and their family caregivers: a controlled study. BMC Cancer. 2007;7:102.

5. Benton MJ, Schlairet MC, Graham HL. Physical activity-related quality of life in breast cancer survivors compared to healthy women. Eur J Cancer Care (Engl). 2019;28(6):e13142.

6. Boehmer U, Ozonoff A, Potter J. Sexual Minority Women's Health Behaviors and Outcomes After Breast Cancer. Lgbt Health. 2015;2(3):221-7.

7. Bøhn SKH, Svendsen K, Balto A, Gjelsvik YM, Myklebust T, Børøsund E, et al. Health-related quality of life among women diagnosed with in situ or invasive breast cancer and age-matched controls: a population-based study. J Patient Rep Outcomes. 2024;8(1):105.

8. Broeckel JA, Jacobsen PB, Balducci L, Horton J, Lyman GH. Quality of life after adjuvant chemotherapy for breast cancer. Breast Cancer Res Treat. 2000;62(2):141-50.

9. Champion VL, Wagner LI, Monahan PO, Daggy J, Smith L, Cohee A, et al. Comparison of younger and older breast cancer survivors and age-matched controls on specific and overall quality of life domains. Cancer. 2014;120(15):2237-46.

10. Chetrit A, Ben-Avraham S, Mandelzweig L, Amitai T, Danieli NS, Silverman B, et al. Breast cancer survivors: physical and mental quality of life 10 years following diagnosis, a case-control study. Breast Cancer Research and Treatment. 2021;188(1):273-82.

11. Claus EB, Petruzella S, Carter D, Kasl S. Quality of life for women diagnosed with breast carcinoma in situ. Journal of Clinical Oncology. 2006;24(30):4875-81.

12. Cordova MJ, Cunningham LLC, Carlson CR, Andrykowski MA. Posttraumatic growth following breast cancer: A controlled comparison study. Health Psychology. 2001;20(3):176-85.

13. de Larrea-Baz NF, Pérez-Gómez B, Guerrero-Zotano A, Casas AM, Bermejo B, Baena-Cañada JM, et al. Primary breast cancer and health related quality of life in Spanish women: The EpiGEICAM case-control study. Scientific Reports. 2020;10(1).

14. Emre N, Yılmaz S. Sleep quality, mental health, and quality of life in women with breast cancer. Indian J Cancer. 2024;61(2):299-304.

15. Fenlon D, Addington-Hall JM, O'Callaghan AC, Clough J, Nicholls P, Simmonds P. A Survey of Joint and Muscle Aches, Pain, and Stiffness Comparing Women With and Without Breast Cancer. Journal of Pain and Symptom Management. 2013;46(4):523-35.

16. Gudbergsson SB, Fosså SD, Sanne B, Dahl AA. A controlled study of job strain in primary-treated cancer patients without metastases. Acta Oncologica. 2007;46(4):534-44.

17. Helgeson VS, Tomich PL. Surviving cancer: a comparison of 5-year disease-free breast cancer survivors with healthy women. Psychooncology. 2005;14(4):307-17.

18. Hermelink K, Voigt V, Kaste J, Neufeld F, Wuerstlein R, Bühner M, et al. Elucidating Pretreatment Cognitive Impairment in Breast Cancer Patients: The Impact of Cancer-Related Post-Traumatic Stress. Jnci-Journal of the National Cancer Institute. 2015;107(7).

19. Hodgson JH, Shields CG, Rousseau SL. Disengaging Communication in Later-Life Couples Coping with Breast Cancer. Families, Systems, & Health. 2003;21(2):145-63.

20. Kang KD, Bae S, Kim HJ, Hwang IG, Kim SM, Han DH. The Relationship between Physical Activity Intensity and Mental Health Status in Patients with Breast Cancer. J Korean Med Sci. 2017;32(8):1345-50.

21. Klein D, Mercier M, Abeilard E, Puyraveau M, Danzon A, Dalstein V, et al. Long-term quality of life after breast cancer: a French registry-based controlled study. Breast Cancer Research and Treatment. 2011;129(1):125-34.

22. Langer D, Tendler S, Bar-Haim Erez A. A broad perspective on breast cancer: Participation, quality of life and return to work throughout the recovery process. Work. 2023;75(1):325-37.

23. Liu S, Wang F, Yang Q, Wang Q, Feng D, Chen Y, et al. Work productivity loss in breast cancer survivors and its effects on quality of life. Work. 2021;70(1):199-207.

24. O'Sullivan MB. Self-perceived health status of Irish breast cancer survivors. Ir J Med Sci. 2001;170(1):14-7.

25. Palomo-López P, Rodríguez-Sanz D, Becerro-de-Bengoa-Vallejo R, Losa-Iglesias ME, Guerrero-Martín J, Calvo-Lobo C, et al. Clinical aspects of foot health and their influence on quality of life among breast cancer survivors: a case-control study. Cancer Management and Research. 2017;9:545-50.

26. Ribeiro IL, Camargo PR, Alburquerque-Sendín F, Ferrari AV, Arrais CL, Salvini TF. Three-dimensional scapular kinematics, shoulder outcome measures and quality of life following treatment for breast cancer - A case control study. Musculoskeletal Science and Practice. 2019;40:72-9.

27. Schleife H, Sachtleben C, Finck Barboza C, Singer S, Hinz A. Anxiety, depression, and quality of life in German ambulatory breast cancer patients. Breast Cancer. 2014;21(2):208-13.

28. Surbhi, Gupta H, Brar G, Jalota V. Quality of life and its sociodemographic determinants in breast cancer patients. Industrial Psychiatry Journal. 2022;31(2):313-7.

29. Tchen N, Juffs HG, Downie FP, Yi QL, Hu H, Chemerynsky I, et al. Cognitive function, fatigue, and menopausal symptoms in women receiving adjuvant chemotherapy for breast cancer. J Clin Oncol. 2003;21(22):4175-83.

30. Tolentino GP, Battaglini CL, Araújo SS, Otano AS, Conde DM, Evans ES, et al. Cardiorespiratory fitness and quality-of-life analysis posttreatment in breast cancer survivors. J Psychosoc Oncol. 2010;28(4):381-98.

31. Tran TXM, Jung SY, Lee EG, Cho H, Kim NY, Shim S, et al. Health-related quality of life in long-term early-stage breast cancer survivors compared to general population in Korea. J Cancer Surviv. 2023.

32. Von Ah DM, Russell KM, Carpenter J, Monahan PO, Qianqian Z, Tallman E, et al. Health-related quality of life of african american breast cancer survivors compared with healthy African American women. Cancer Nurs. 2012;35(5):337-46.

33. Yabroff KR, McNeel TS, Waldron WR, Davis WW, Brown ML, Clauser S, et al. Health limitations and quality of life associated with cancer and other chronic diseases by phase of care. Med Care. 2007;45(7):629-37.

34. Yu J, Son WS, Lee SB, Chung IY, Son BH, Ahn SH, et al. Uneven recovery patterns of compromised health-related quality of life (EQ-5D-3 L) domains for breast Cancer survivors: a comparative study. Health Qual Life Outcomes. 2018;16(1):143.

35. Zhang JH, Zhu YS, Zhang Y. Correlation of Social Support and Quality of Life in Patients with Mammary Cancer. Journal of contemporary nurse. 2011(12):1-3.

36. Zhang FY, Tong JC. A correlative study on social support and quality of life of breast cancer patients. Chinese Nursing Research. 2008;22(18):1606-7.
